# Preparation of Biochars from Different Sources and Study on Their Phosphorus Adsorption Properties

**DOI:** 10.3390/molecules30122633

**Published:** 2025-06-18

**Authors:** Yinlong Shao, Anqi Hu, Yongcan Jiang, Xianbiao Wang, Jingchen Li, Guanglong Liu

**Affiliations:** 1 PowerChina Huadong Engineering Corporation Ltd., Hangzhou 311122, China; shao_yl@hdec.com (Y.S.);; 2 Key Laboratory of Arable Land Conservation (Middle and Lower Reaches of Yangtze River), Ministry of Agriculture and Rural Affairs, College of Resources and Environment, Huazhong Agricultural University, Wuhan 430070, China; 3 Institute of Soil and Water Resources and Environmental Science, College of Environmental and Resource Sciences, Zhejiang University, Hangzhou 310058, China; 4 College of Landscape and Horticulture, Hubei Vocational College of Bio-Technology, Wuhan 430070, China

**Keywords:** biochar, modification, adsorption, phosphorus, iron oxide

## Abstract

Biochar is a solid product generated through the pyrolysis of biomass materials under anaerobic or hypoxic conditions, and it is characterized by its strong adsorption capacity. To investigate the phosphorus adsorption performance of biochar derived from wheat straw, bamboo, and water hyacinth in wastewater, iron modification treatments were applied to these biochars, and the most effective modified biochar was identified. The physicochemical properties of the modified biochars were characterized using Fourier Transform Infrared Spectroscopy (FTIR), X-ray Diffraction (XRD), and scanning electron microscopy (SEM). The results showed that optimal modification was achieved with an iron–carbon mass ratio of 0.70 for wheat straw biochar (Fe-WBC) and 0.45 for both bamboo biochar (Fe-BBC) and water hyacinth biochar (Fe-HBC). The maximum phosphorus adsorption capacities of the three modified biochars were as follows: 31.76 mg g^−1^ (Fe-WBC) > 27.14 mg g^−1^ (Fe-HBC) > 25.31 mg g^−1^ (Fe-BBC). It was demonstrated that the adsorption behavior of Fe-BBC was predominantly multi-molecular layer adsorption, whereas the adsorption behavior of Fe-WBC and Fe-HBC was primarily monolayer adsorption. All three types of modified biochars reached adsorption equilibrium within 30 min, with Fe-WBC exhibiting the best adsorption performance. Analysis revealed that the modified biochars contained a large number of unsaturated C bonds and aromatic rings, indicating relatively stable structures. The surfaces of the modified biochars were rich in hydroxyl and carbonyl groups, which contributed to their strong adsorption properties. Post-modification analysis indicated that iron in the biochars predominantly existed in forms such as goethite (FeOOH) and hematite (Fe_2_O_3_). The iron content in each type of modified biochar constituted approximately 3.08% for Fe-WBC, 5.94% for Fe-BBC, and 5.68% for Fe-HBC relative to their total elemental composition. Overall, the iron-modified biochars employed in this study significantly enhanced the adsorption capacity and efficiency for phosphorus removal in wastewater.

## 1. Introduction

Phosphorus (P), an essential chemical element, is widely used in fertilizers, detergents, corrosion inhibitors, and various pharmaceuticals, and it is continuously discharged into both industrial and municipal wastewater streams [1,2]. The release of significant nutrient elements, such as nitrogen (N) and phosphorus, into water bodies via multiple pathways leads to water eutrophication [3,4]. This phenomenon triggers the rapid proliferation of algae and microscopic plankton, which consume substantial amounts of dissolved oxygen (O_2_) during decomposition, causing mass mortality of fish and other aquatic organisms [5,6]. Eutrophication poses a severe threat to aquatic biodiversity and can lead to ecological imbalances [7,8,9]. Moreover, phosphorus is a finite resource primarily obtained through mining and is classified as non-renewable [10]. Given the high demand for phosphorus, particularly in the context of rapid industrial and agricultural development, excessive mining has led to a sharp decline in phosphate resources. Therefore, recovering phosphorus from wastewater is critical for addressing phosphorus scarcity [11].

Currently, the methods for phosphorus removal in wastewater treatment include chemical precipitation, biological processes, and adsorption techniques [12,13,14]. Among these, the adsorption method is characterized by its low cost, renewability, environmental friendliness, and high efficiency [15,16,17]. This method employs loose, porous solid adsorbents with a large specific surface area to adsorb phosphate ions from water, thereby enabling both phosphorus removal and recovery from wastewater [18]. In recent years, biochar, an economical and environmentally friendly material, has been widely applied in the remediation of phosphorus pollution in aquatic environments [19,20,21]. Moreover, its applications are expanding beyond environmental remediation, with various applications of biochar in sensing [22], biosensing [23], and energy storage. Biochar refers to a highly aromatic, structurally porous, and insoluble solid substance formed through the pyrolysis and carbonization of waste organic biomass under oxygen-free or oxygen-limited conditions at temperatures below 700 °C [24]. Variations in raw materials and pyrolysis conditions result in significant differences in biochar’s pore structure, specific surface area, surface oxygen-containing functional group content, and elemental composition, which in turn influence its performance and mechanisms in various applications [25].

Common biochar raw materials include charcoal, plant shell charcoal, straw charcoal, and animal dung charcoal [26,27]. However, natural biochar exhibits a relatively simple pore structure, a smaller specific surface area, and limited adsorption capacity [28,29]. Modification represents an effective approach to enhance the adsorption, activation, and physicochemical properties of biochar for phosphate removal [30,31,32]. A common modification technique involves the incorporation of metal cations into biochar [33,34]. Biochar modified with metal oxides or metal salts demonstrates superior electrostatic force, precipitation capability, and anion exchange capacity, thereby significantly improving phosphorus adsorption performance [35,36,37]. Liu et al. (2022) modified biochar with La(OH)₃, achieving nearly 100% phosphorus adsorption efficiency in water [7]. Liu et al. (2024) developed a novel calcium-modified biochar (MBC) using waste coffee grounds and CaCl_2_ as raw materials and modifiers, respectively, with a maximum adsorption capacity of 70.26 mg g^−1^ [10]. The MGO-biochar nanocomposites prepared by Zhang et al. (2012) exhibited an adsorption capacity as high as 835 mg g^−1^ [37]. fabricated nano CaO_2_/BC composites that demonstrated excellent phosphorus adsorption performance in water, with a capacity of 213.22 ± 13.57 mg g^−1^ [38].

Every year, a significant portion of wheat straw is directly incinerated, with only a minor fraction utilized for field return, papermaking, and livestock feeding, leading to energy wastage and environmental pollution [39]. Wheat straw biochar, produced through thermal cracking, can serve as an effective adsorbent for remediating water pollution [40,41]. Water hyacinth, a common aquatic plant, exhibits robust growth and reproduction capabilities, frequently causing large-scale outbreaks that severely impact aquaculture, aquatic biodiversity, and water quality [42]. Consequently, utilizing biochar derived from water hyacinth as an adsorbent for water pollutant removal represents a practical approach to achieving resource utilization while mitigating the adverse effects of invasive plants on aquatic ecosystems and economic development [43]. Bamboo carbonized at temperatures between 600 °C and 700 °C undergoes rapid micropore expansion, forming bamboo charcoal biochar with a pore structure resembling onion-like C60 composed of five- and six-membered rings and expanded carbon nanotube structures. This unique pore architecture endows bamboo charcoal biochar with excellent mechanical properties, favorable processability, and superior material transport capacity [44,45]. The specific surface area of typical bamboo biochar can reach up to 360 m^2^ g^−1^, making it an outstanding adsorbent [46,47]. Furthermore, iron oxides are ubiquitously present in soils and sediments [48]. Iron oxides exhibit strong activity and thermal stability, possess a large specific surface area, and demonstrate excellent oxidation–reduction properties, playing a crucial role in natural phosphorus purification processes [49,50,51]. Therefore, this study prepared biochar from three raw materials—wheat straw, bamboo, and hyacinth—and modified it by iron loading to develop a highly efficient phosphorus adsorbent. The adsorption performance of the iron-modified biochar for phosphate removal in water was investigated, and the superior biochar was characterized to elucidate the reasons for its exceptional adsorption capacity.

The objectives of this study were twofold: (1) To develop iron-modified biochars from wheat straw, bamboo, and hyacinth via chemical modification, with a focus on loading iron onto the biochar matrix to enhance its functional properties. (2) Based on the phosphorus adsorption capacity of the modified biochar, the material exhibiting the highest phosphorus adsorption capacity was identified as the optimal modified biochar. Subsequently, the predominant modified biochar for each material was selected to investigate its phosphorus adsorption characteristics. The effects of initial phosphorus concentration and adsorption time on the adsorption properties of the three types of modified biochar were also examined. Additionally, the physical and chemical properties of the biochar, including morphology, elemental composition, crystalline phase structure, and surface functional groups, were comprehensively analyzed.

## 2. Results and Discussion

### 2.1. Determination of Modified Biochar

**Figure 1:**
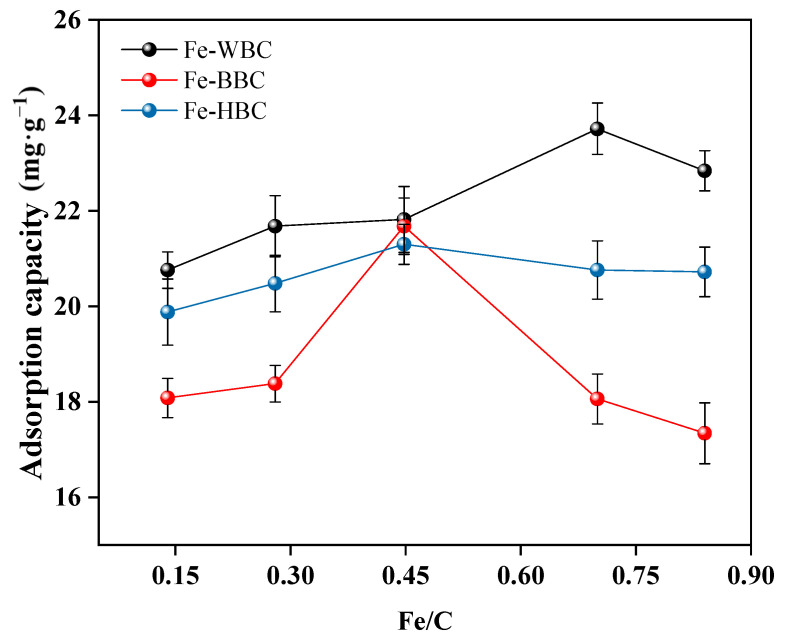
Comparison of phosphorus adsorption capacity of modified biochar with different iron–carbon ratios.

The phosphorus adsorption capacities of the three raw materials subjected to different modifications are shown in Figure 1. As the iron-to-carbon ratio increases, the phosphorus adsorption capacity of iron-modified biochar initially rises and then decreases. The maximum phosphorus adsorption capacity of modified wheat straw biochar was 23.72 mg g^−1^ at an iron-to-carbon ratio of 0.70. For modified bamboo charcoal biochar and hyacinth biochar, the maximum phosphorus adsorption capacities were 21.68 mg g^−1^ and 21.35 mg g^−1^, respectively, at an iron-to-carbon ratio of 0.45. Therefore, the modified wheat straw biochar with an iron-to-carbon ratio of 0.70 was designated as Fe-WBC, while the modified bamboo charcoal biochar and hyacinth biochar with an iron-to-carbon ratio of 0.45 were designated as Fe-BBC and Fe-HBC, respectively. These optimally modified biochars were selected for subsequent experiments.

### 2.2. Influence of Initial Concentration on Phosphorus Adsorption

Under specific temperature conditions, the adsorption of phosphorus by modified biochar is a dynamic equilibrium process. When adsorption reaches equilibrium, the relationship between the phosphorus adsorption capacity of the modified biochar and the equilibrium concentration of the phosphorus solution can be described using an adsorption isotherm [52,53]. In this study, the Langmuir and Freundlich adsorption models were employed to fit the experimental data, thereby facilitating the analysis of the influence of initial phosphate concentration on phosphorus adsorption by biochar. According to the Langmuir adsorption model, the adsorbent surface is considered a single-layer homogeneous surface, and the adsorption behavior on this surface involves monomolecular layer adsorption without mutual interactions between adsorbed molecules [54,55]. Furthermore, when the adsorbent surface becomes saturated, the adsorption capacity reaches its maximum value. The Freundlich isothermal adsorption model is an empirical formula typically employed to describe multi-molecular layer adsorption with complex adsorption behaviors occurring on heterogeneous surfaces. This model is applicable to various physical and chemical conditions [56].

**Figure 2:**
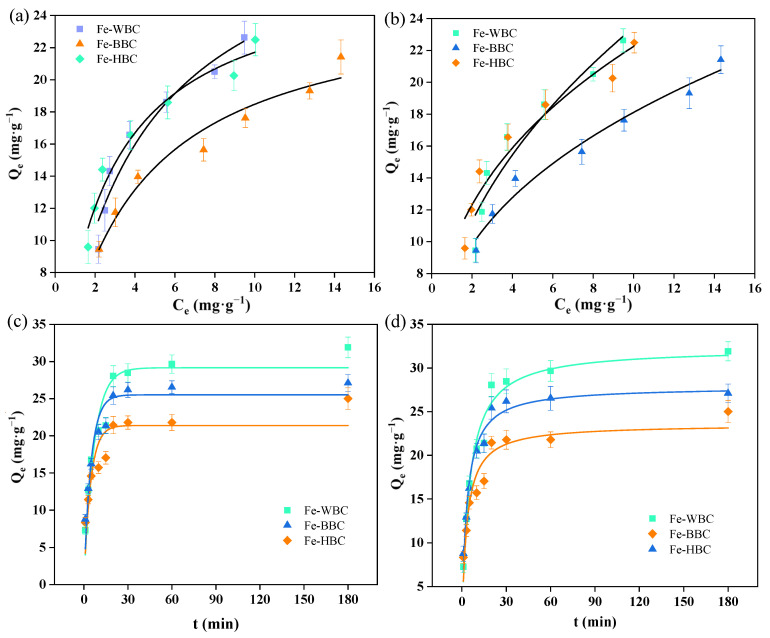
Langmuir (**a**) and Freundlich (**b**) isothermal adsorption lines and pseudo-first-order (**c**) and pseudo-second-order (**d**) kinetic fitting curves for phosphorus of three modified biochar.

The Langmuir and Freundlich adsorption isotherms for phosphorus of Fe-WBC, Fe-BBC, and Fe-HBC are presented in Figure 2a and Figure 2b, respectively. As the equilibrium concentration of the phosphorus solution increases, the adsorption capacity of the modified biochars also increases. With further increases in concentration, the adsorption capacity tends to stabilize, eventually reaching adsorption equilibrium. Based on the fitting results of the Langmuir adsorption model, the maximum adsorption capacities were determined as follows: 31.76 mg g^−1^ for Fe-WBC, 25.31 mg g^−1^ for Fe-BBC, and 27.14 mg g^−1^ for Fe-HBC. These results indicate that among the three types of biochar derived from different raw materials, Fe-WBC exhibits the highest adsorption capacity, significantly surpassing the modified carbons prepared from the other two materials. Fe-HBC ranks second in terms of adsorption capacity, while Fe-BBC demonstrates the lowest adsorption capacity.

As shown in Table 1, the R^2^ values obtained by fitting Fe-WBC, Fe-BBC, and Fe-HBC with the Langmuir isothermal adsorption equation were 0.948, 0.960, and 0.965, respectively. Meanwhile, the R^2^ values obtained by fitting the three modified biochars with the Freundlich adsorption equation were 0.921, 0.975, and 0.938, respectively. The results indicate that the Freundlich adsorption equation better describes the phosphorus adsorption characteristics of Fe-BBC, suggesting that the adsorption behavior of Fe-BBC is predominantly multi-molecular layer adsorption. In contrast, the Langmuir isothermal adsorption equation provides a better description of the adsorption characteristics of Fe-WBC and Fe-HBC, indicating that their adsorption behavior is primarily monolayer adsorption [57,58,59]. In the Langmuir adsorption equation, the parameter K_L_ can be utilized to represent the binding stability between modified biochar and phosphorus [60]. When K_L_ > 1, the reaction corresponds to unfavorable adsorption. Favorable adsorption occurs when 0 < K_L_ < 1, while irreversible adsorption is indicated when K_L_ = 0. A larger K_L_ value signifies stronger binding stability between the adsorbent and the adsorbate. As shown in Table 1, the K_L_ values of the three modified biochars all fall within the range of 0 to 1, indicating that the adsorption process is favorable. The K_L_ values follow the order Fe-HBC > Fe-BBC > Fe-WBC, suggesting that the binding stability between Fe-HBC and phosphorus is the strongest, whereas that between Fe-WBC and phosphorus is the weakest. In the Freundlich adsorption equation, the slope 1/n represents the difficulty of the adsorption process [61]. When 0.1 < n < 1, the adsorption process is considered favorable. A smaller value of 1/n indicates a stronger interaction between the adsorbent and the adsorbate [21]. As shown in Table 1, the 1/n values of the three modified biochars fall within the range of 0.1 to 1, suggesting that the adsorption process is favorable. Notably, the 1/n value of Fe-HBC is the smallest, indicating its strongest binding ability with phosphorus.

### 2.3. Influence of Reaction Time on Phosphorus Adsorption

Adsorption kinetics can be employed to describe the rate at which biochar adsorbs phosphorus [62]. In this study, both the pseudo-first-order kinetic equation and the pseudo-second-order kinetic equation were utilized to fit the experimental data. This approach enabled an investigation into the kinetics of phosphorus adsorption by modified biochar and facilitated an analysis of the influence of reaction time on phosphorus adsorption by Fe-WBC, Fe-BBC, and Fe-HBC. The pseudo-first-order and pseudo-second-order kinetic adsorption curves for phosphorus adsorption by Fe-WBC, Fe-BBC, and Fe-HBC are presented in Figure 2c,d. As shown in the figure, the trends of phosphorus adsorption by modified biochars derived from different raw materials are similar. With increasing reaction time, the phosphorus adsorption capacity of the modified biochars initially increases and subsequently tends toward equilibrium. Fe-WBC reached adsorption equilibrium at approximately 25 min, with an adsorption capacity of 32.29 mg g^−1^. Fe-BBC achieved equilibrium at 20 min, exhibiting an adsorption capacity of 27.86 mg g^−1^. Fe-HBC attained equilibrium at 15 min, with an adsorption capacity of approximately 23.54 mg g^−1^. It can be observed that when the reaction time is less than 30 min, the rate of phosphorus adsorption by the modified biochars is relatively fast, and the adsorption capacity continues to increase. After 30 min of adsorption, the increase in adsorption capacity slows down, indicating that the phosphorus adsorption process by biochar is rapid. Upon reaching adsorption equilibrium, the phosphorus adsorption capacities of the three modified biochars followed the order Fe-WBC > Fe-HBC > Fe-BBC, further confirming that Fe-WBC exhibited the best adsorption performance.

Kinetics was employed to investigate the control mechanisms in the adsorption process, and the phosphorus adsorption kinetics of iron-modified biochar derived from three different raw materials were analyzed. The fitting parameters obtained from the experimental data are presented in Table 2. Comparing the fitting parameters of the two adsorption models, it can be observed that the R^2^ values for the pseudo-second-order kinetic adsorption models of Fe-WBC, Fe-BBC, and Fe-HBC are 0.9633, 0.9628, and 0.8915, respectively, all of which exceed those of the pseudo-first-order kinetic model. Therefore, the pseudo-second-order kinetic model provides a better description of the adsorption kinetics of phosphorus by the three types of modified biochar. It has been reported that the pseudo-first-order kinetic model is associated with the concentration of the adsorbate in the bulk solution, whereas the pseudo-second-order kinetic model relates to the availability of active sites on the surface of the adsorbent [63]. The pseudo-second-order kinetic model posits that the adsorption driving force is proportional to the square of the adsorption rate, enabling it to describe both the fast and slow stages of adsorption and more accurately reflect the overall adsorption process [64]. The results indicate that the adsorption of phosphorus by Fe-WBC, Fe-BBC, and Fe-HBC is primarily chemical in nature, with the adsorption process predominantly occurring on the biochar surface [65]. After Fe modification, the biochar surface becomes enriched with iron oxides, and the hydroxyl functional groups on the iron oxide surface react with phosphoric acid, thereby immobilizing phosphorus onto the adsorbent material and achieving the goal of phosphorus removal. Based on the aforementioned analysis, it can be concluded that the three modified biochars effectively adsorb substantial amounts of phosphorus, primarily through surface adsorption.

### 2.4. Characterization of Biochar

#### 2.4.1. FTIR Spectra of Biochar

**Figure 3:**
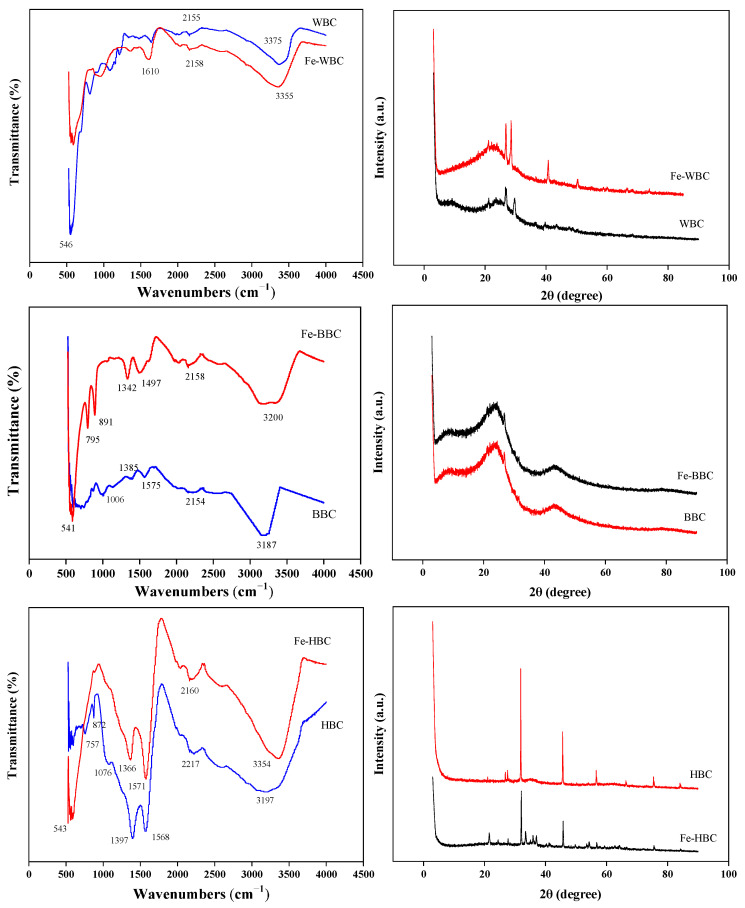
FTIR and XRD spectra of three kinds of biochar before and after modification.

As shown in Figure 3, the infrared spectra of the three biochars reveal that the main absorption peaks of WBC and Fe-WBC appear near 3350 cm^−1^ and 2160 cm^−1^. Typically, broad peaks within the wavenumber range of 3000–3700 cm^−1^ are attributed to hydroxyl (–OH) stretching vibrations [66]. The absorption peak at wavenumbers of 1570–1610 cm^−1^ is associated with the stretching vibrations of C=O and C=C bonds [67,68]. Consequently, the broad absorption peaks of WBC and Fe-WBC near 3350 cm^−1^ were identified as –OH stretching vibration peaks, which may originate from intermolecular hydrogen bonds of carbohydrates in organic compounds. Meanwhile, the absorption peaks of WBC and Fe-WBC near 2160 cm^−1^ correspond to the stretching vibrations of alkyne C. Additionally, the absorption peak of Fe-WBC at 1616 cm^−1^ represents the stretching vibration of C=C and C=O groups in aromatic rings. Furthermore, the absorption peak of Fe-WBC at 546 cm^−1^ corresponds to the stretching vibration of Fe-O. The broad absorption peaks of BBC and Fe-BBC near 3200 cm^−1^ and 2160 cm^−1^ are attributed to the stretching vibrations of the hydroxyl group (–OH) and alkyne C, respectively. The absorption peaks in the range of 1340–1465 cm^−1^ are associated with the bending vibrations of saturated alkane C–H bonds. The absorption peak of BBC at 1575 cm^−1^ corresponds to the stretching vibrations of C=C and C=O groups in aromatic rings. Additionally, the C–O stretching vibration peak of alcohol appears in BBC at 1006 cm^−1^. For Fe-BBC, the absorption peaks at 795 cm^−1^ and 891 cm^−1^ correspond to the out-of-plane bending vibrations of aromatic ring C–H. Furthermore, a new absorption peak for Fe-BBC at 541 cm^−1^ is observed, which is attributed to the stretching vibration of Fe–O in hematite. The absorption peaks of HBC and Fe-HBC at 3197 cm^−1^ and 3354 cm^−1^, respectively, correspond to the stretching vibration peaks of –OH. The absorption peaks at 2217 cm^−1^ and 2160 cm^−1^ are attributed to the stretching vibrations of alkyne C. The strong absorption peaks of Fe-HBC and HBC near 1570 cm^−1^ are associated with the stretching vibrations of C=C and C=O in aromatic rings. The prominent peaks between 1310 cm^−1^ and 1410 cm^−1^ may result from the in-plane deformation vibrations of –OH groups in tertiary alcohols. The C–O stretching vibration peak of alcohol is observed in HBC at 1076 cm^−1^, and the out-of-plane bending vibrations of aromatic ring C–H occur in HBC at 872 cm^−1^ and 757 cm^−1^. Compared with HBC, Fe-HBC exhibits a stretching vibration peak corresponding to hematite Fe–O at 543 cm^−1^. As shown by the infrared spectra, most carbons in the three biochars and their modified forms exist as unsaturated C bonds and aromatic ring structures, indicating relatively stable configurations. The abundant functional groups, such as hydroxyl and carbonyl groups, on the biochar surfaces confirm the strong adsorption properties of the biochars. Additionally, the presence of Fe–O demonstrates that the biochars are successfully loaded with iron oxides.

#### 2.4.2. XRD Analysis of Biochar

As shown in the XRD patterns of the three biochars presented in Figure 3, the diffraction peak corresponding to 2θ = 20°–24° represents unorganized carbon, which is observed in WBC, BBC, HBC, Fe-WBC, Fe-BBC, and Fe-HBC. Due to strong acid treatment and iron loading, the peak intensity of the modified carbons derived from the three different raw materials is weakened. However, the diffraction peak at 2θ = 27.5°–29° is enhanced, indicating an increase in the degree of graphitization of the biochar. The diffraction peaks of the three modified biochars at 2θ = 32° correspond to goethite (FeOOH). The diffraction peak of Fe-HBC at 2θ = 53.1° also corresponds to goethite (FeOOH) [69]. The diffraction peaks of Fe-WBC at 2θ = 41.0° and 49.6°, Fe-BBC at 2θ = 43.6°, and Fe-HBC at 2θ = 46.4° and 57.8° correspond to hematite (Fe_2_O_3_) [70]. Additionally, the diffraction peaks of Fe-WBC and Fe-HBC at 2θ = 76.5° are consistent with those of ferrous oxide (FeO). The XRD results indicate that the primary products formed during the iron-loading process in this experiment are goethite (FeOOH) and hematite (Fe_2_O_3_). Furthermore, the presence of FeO crystals in the modified carbons may result from the mutual conversion of FeOOH and FeO under specific conditions, demonstrating the complexity of iron oxide transformations during the iron-loading modification process and the difficulty of obtaining a single product.

#### 2.4.3. SEM and EDS Analysis of Biochar

**Figure 4:**
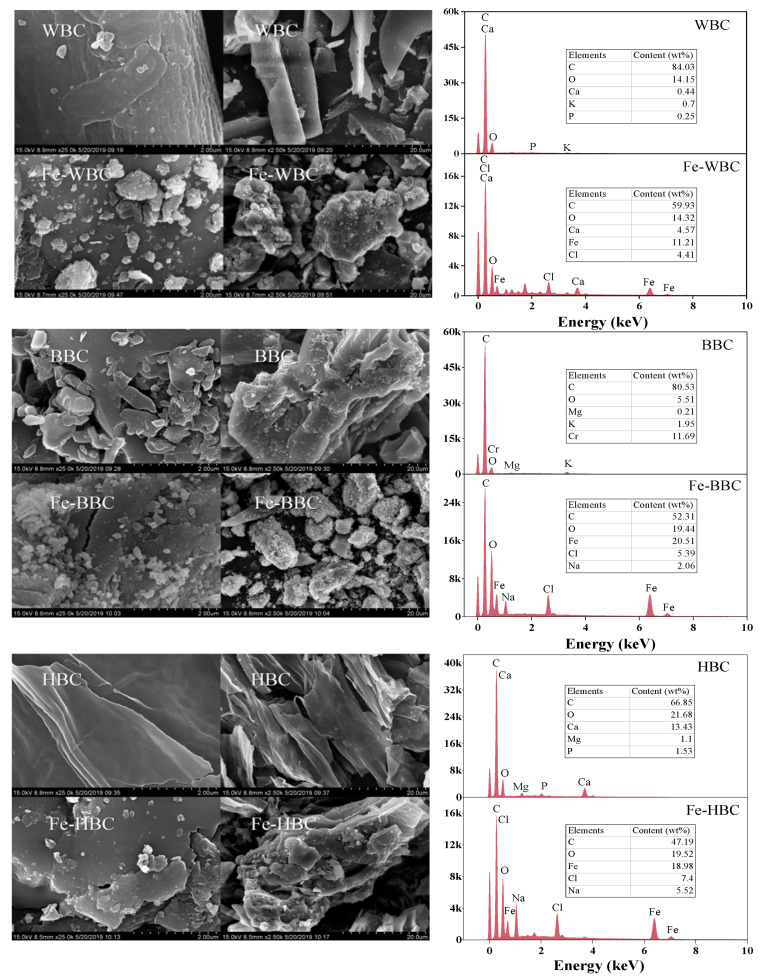
SEM and EDS spectra of three kinds of biochar before and after modification.

As shown in Figure 4, WBC exhibited a stacked lamellar structure before modification, featuring numerous micropores on the surface, thin pore walls, and a small amount of clastic particulate matter, which may correspond to crystalline particles formed by ash elements such as Ca and K. The stacking of the lamellar structure could be attributed to the exothermic reaction of wheat straw during pyrolysis, where a significant amount of energy is released from within the pores. This reorganizes the lamellar structure, leading to a disordered distribution of pores. Despite this, pore structures still exist around the pore walls of the lamellar stacking, contributing to its high adsorption capacity. After modification, the surface of Fe-WBC becomes rougher with irregular granular crystals attached. According to the EDS spectrum, Fe atoms account for 3.08% of the total elemental composition of Fe-WBC. The surface of the modified BBC is smooth, with numerous large crystals distributed across it. These crystals exhibit irregular shapes and vary in size. After modification, small circular pores appear on the surface of Fe-BBC, rendering its surface rougher. This unique structure enhances the specific surface area of Fe-BBC and provides additional multiple adsorption sites. Consequently, it can be inferred that the modification significantly improves the adsorption performance of Fe-BBC. Furthermore, the EDS spectrum of Fe-BBC confirms that the modified biochar is successfully loaded with Fe, with its atomic content accounting for approximately 5.94% of the total elemental composition. The special structure of HBC prior to modification renders its surface smooth and compact, with abundant pores exhibiting a scattered distribution. At 550 °C, most organic compounds comprising HBC are decomposed by high temperature, forming an aromatic ring structure. However, the morphological features of HBC after modification differ significantly from those before modification. Numerous newly formed flake-like crystals are distributed on the surface and within the pores of HBC, increasing the irregularity of the biochar surface. This indicates that iron has been successfully loaded onto the biochar. Furthermore, the EDS spectrum of Fe-HBC confirms that the modified biochar contains Fe, which is consistent with the aforementioned conclusion. The atomic content of Fe accounts for approximately 5.68% of the total elemental composition.

EDS analysis revealed distinct iron contents among the modified biochars: 3.08% for Fe-WBC, 5.94% for Fe-BBC, and 5.68% for Fe-HBC. Notably, Fe-WBC exhibited the highest phosphorus adsorption capacity (31.76 mg/g) despite its lower iron loading, indicating that adsorption performance is not directly governed by iron content alone. XRD results showed that Fe-WBC predominantly formed hematite (Fe_2_O_3_), while Fe-BBC and Fe-HBC contained higher proportions of goethite (FeOOH). The crystalline hematite in Fe-WBC likely enabled stronger monolayer adsorption (Langmuir R^2^ > 0.94), supported by FTIR-detected hydroxyl groups (3350 cm^−1^) that facilitate phosphate complexation. In contrast, the amorphous goethite in Fe-BBC and Fe-HBC favored multi-layer adsorption (Freundlich R^2^ = 0.975 for Fe-BBC), though their higher iron contents did not surpass Fe-WBC’s capacity. These findings highlight that optimizing iron oxide phases (e.g., hematite/goethite ratio) is more critical than increasing iron loading for enhancing adsorption efficiency.

In this study, the modified biochar material was characterized based on its physicochemical properties, revealing that the iron oxides loaded onto the biochar primarily existed in the forms of goethite (α-FeOOH) and hematite (α-Fe_2_O_3_). Ristić et al. (2015) proposed that α-FeOOH and α-Fe_2_O_3_ exhibit a competitive formation mechanism in alkaline media [71]. Under alkaline conditions, the co-precipitation reaction between FeCl_3_ solution and NaOH predominantly yields α-FeOOH and α-Fe_2_O₃ as the main products. The findings of this experiment are largely consistent with this conclusion. Both α-FeOOH and α-Fe_2_O_3_ possess high surface activity and specific surface area, which contribute to their excellent phosphorus adsorption performance. Consequently, the FeCl_3_-based modification method employed in this study significantly enhances the phosphorus adsorption capacity and efficiency of the biochar. The stable embedding of iron oxides into the biochar matrix (XRD/SEM-EDS) and surface functional groups (FTIR) reduces the risk of leaching, but the long-term stability under different pH conditions needs to be further studied.

## 3. Materials and Methods

### 3.1. Preparation of Raw Biochar

In this experiment, wheat straw biochar and bamboo charcoal biochar were procured from Shanghai Vita Chemical Reagent Co., Ltd. (Shanghai, China) as the raw material biochars for wheat straw and bamboo. Hyacinth biochar was utilized as the raw material in the constructed wetland at Huazhong Agricultural University. After natural air-drying, the biochars were crushed to ≤5 mm, washed 2–3 times with ultrapure water (UP), dried, and subsequently placed in a box-type resistance furnace (SX2-8-10, Shanghai Yiheng Scientific Instrument Co., Ltd., Shanghai, China). The temperature was increased to 550 °C at a rate of 12.50 °C/min, followed by cooling to room temperature at the set temperature. The samples were then sealed and stored. The raw materials of wheat straw biochar, bamboo biochar, and hyacinth biochar were labeled as WBC, BBC, and HBC, respectively.

### 3.2. Preparation and Comparison of Modified Biochar

Three raw material biochars of 100 g were soaked in 1 L 1 mol/L of HCL for 1 h, and then pumped and filtered, and the pH of the filtrate was determined with pH precision test paper until the filtrate was neutral. The raw biochar after extraction was dried in an electric blast drying oven at 75 °C and sealed. Weigh a certain amount of biochar soaked in hydrochloric acid and then dried, add the biochar into a certain amount of 1 mol/L FeC1_3_ solution according to different iron–carbon mass ratios (0.140, 0.280, 0.448, 0.700, 0.840), and adjust the mixture with 5 mol/L NaOH solution to a pH value of 11~13. Stir thoroughly until evenly mixed, stand for 1 h, centrifuge, pour out the supernatant, and bake the remaining mixture in an electric blast drying oven at 75 °C to constant weight to obtain iron-modified biochar.

Under indoor temperature conditions, 0.2 g of the above modified biochar was taken into a 100 mL centrifuge tube, then 50 mL of KH_2_PO_4_ solution with a concentration of 50 mg/L was added and centrifuged at a temperature of 25 °C for 120 r/min for 3 h. After filtration by a 0.45 μm aqueous filter, the concentration of phosphorus in the filtrate was determined by spectrophotometry. Set three parallels for each processing group. According to the formula (Equation (1)), the adsorption capacity was calculated, and the modified biochar with the largest adsorption capacity was selected as the best modified biochar, labeled as Fe-WBC, Fe-BBC, and Fe-HBC.

(1) $$q_{e}=\frac{V}{m}\left(C_{0}-C_{e}\right)\;$$

where *q_e_* is the adsorption amount at adsorption equilibrium (mg/g); *C*_0_ is the initial solution concentration (mg/L); *C_e_* is the solution concentration at adsorption equilibrium (mg/L); V is the solution volume (L); and m is the amount of adsorbent (g).

### 3.3. Adsorption Experiment

#### 3.3.1. Adsorption Isotherm Experiment

Fe-WBC, Fe-BBC, and Fe-HBC selected by 0.2 g ratio were, respectively, weighed into a 100 mL centrifuge tube, and 50 mI KH_2_PO_4_ solution with concentrations of 40, 50, 60, 70, 80, 90, and 100 mg/L were added, respectively. After shaking at 25 °C at 120 r/min for 3 h, centrifugation and filtration by a 0.45 μm aqueous filter membrane were performed to determine the concentration of phosphorus in the filtrate. Set three parallels for each processing group. Two models, *Langmuir* (Equation (2)) and *Freundlich* (Equation (3)), were used to simulate adsorption isotherms.

(2) $$Q_{e}=\frac{K_{L}Q_{m}C_{e}}{1+K_{L}C_{e}}\;$$

(3) $$Q_{e}=K_{F}C_{e}^{\frac{1}{n}}\;$$

where *Q_e_* (mg P/g) is the amount of phosphate adsorbed at the equilibrium concentration of phosphate solution (C*_e_*, mg P/L), K*_L_* and K*_F_* are *Langmuir* constant and *Freundlich* constant, respectively, *Q_m_* (mg P/g) is the maximum adsorption capacity, and 1/n is the empirical constant of the *Freundlich* model.

#### 3.3.2. Adsorption Kinetics Experiment

Fe-WBC, Fe-BBC, and Fe-HBC selected by 0.2 g ratio were, respectively, weighed into a 100 mL centrifuge tube, and KH_2_PO_4_ solution with a concentration of 50 mg/L was added at 25 °C and a rotating speed of 120 r/min. After shaking for 5, 10, 15, 20, 30, 60, 120, and 180 min and centrifugation, with filtration by a 0.45 μm aqueous filter membrane, the concentration of phosphorus in filtrate was determined. Set three parallels for each processing group. Quasi-first-order (Equation (4)) and quasi-second-order (Equation (5)) were used to determine the adsorption kinetics of phosphate by modified biochar.

(4) $$Q_{t}=Q_{e}\left(1-\exp\left(-K_{1}t\right)\right)$$

(5) $$Q_{t}=\frac{K_{2}Q_{e}^{2}t}{1+K_{2}Q_{e}t}$$

where *K*_1_ (min^−1^) and *K*_2_ are the rate constants of the pseudo-first-order and pseudo-second-order models, respectively, *Q_t_* (mg P/g) is the amount of phosphate adsorbed at time *t*, and *Q_e_* (mg P/g) is the amount of phosphate adsorbed at equilibrium state.

### 3.4. Material Characterization

A Fourier transform infrared spectrometer (FT-IR, Bruker VERTEX70, Billerica, MA, USA) was used to analyze and determine the infrared spectrum of modified biochar. Biochar and KBr were mixed and compressed in a ratio of 1:20, with a scanning range of 400~4000 cm^−1^ and a resolution of 4 cm^−1^. An X-ray diffractometer (XRD, Bruker D8 Advance X, Wolzbach, Germany) was used to analyze the crystal phase structure of biochar materials with a scanning range of 3°~90° and a scanning rate of 10°·min^−1^. The surface morphology and elemental composition of the biochars were characterized by a scanning electron microscopy–energy dispersion spectrometer (SEM-EDS, Hitachi SU8020, Tokyo, Japan).

## 4. Conclusions

In this experiment, biochars derived from wheat straw, bamboo, and hyacinth raw materials were selected for modification with Fe^3^⁺, and the adsorption performance of the modified biochars on phosphorus in water bodies was investigated. When the optimal mixed mass ratios of Fe^3^⁺ to wheat straw biochar (Fe-WBC), bamboo biochar (Fe-BBC), and hyacinth biochar (Fe-HBC) were 0.70, 0.45, and 0.45, respectively, the phosphorus adsorption capacities of the modified biochars reached their highest values. Based on the fitting results of the Langmuir adsorption model, the maximum adsorption capacities were determined as follows: 31.76 mg g^−1^ for Fe-WBC, 25.31 mg g^−1^ for Fe-BBC, and 27.14 mg g^−1^ for Fe-HBC. According to the results of the adsorption isotherm analysis, it was demonstrated that the adsorption behavior of Fe-BBC was predominantly multi-molecular layer adsorption, whereas the adsorption behavior of Fe-WBC and Fe-HBC was primarily monolayer adsorption. Kinetic adsorption experiments revealed that the adsorption of phosphorus by Fe-WBC, Fe-HBC, and Fe-BBC mainly relied on chemical adsorption, and the adsorption process reached equilibrium within 30 min. The physicochemical properties of Fe-WBC, Fe-HBC, and Fe-BBC were characterized, and it was found that the modified biochars contained a large number of unsaturated C bonds and aromatic rings, indicating relatively stable structures. The surfaces of the modified biochars were rich in hydroxyl and carbonyl groups, which contributed to their strong adsorption properties. The iron supported on the iron-modified biochars primarily existed in the forms of goethite (FeOOH) and hematite (Fe_2_O_3_). The Fe content supported on the three modified biochars accounted for 3.08% (Fe-WBC), 5.94% (Fe-BBC), and 5.68% (Fe-HBC) of their total elemental compositions, respectively. Iron-modified biochar can be applied to phosphorus-containing wastewater treatment, addressing solid waste disTokyovposal while enabling phosphorus recovery. This integrated approach not only highlights the potential of iron-modified biochars for large-scale, sustainable wastewater treatment and resource management but also provides a practical solution for phosphorus removal at the macro-scale, validated by pilot-scale feasibility and aligned with circular economy principles.

## Figures and Tables

**Table 1:** Simulation of isothermal adsorption model and corresponding parameters.

Modified Biochar	Langmuir Parameters			Freundlich Parameters		
	K_L_ (L mg^−1^)	Q_m_ (mg g^−1^)	R^2^	K_F_ (mg g^−1^)(L·mg^−1^)^1/n^	n	R^2^
Fe-WBC	0.253	31.76	0.948	8.212	2.195	0.921
Fe-BBC	0.270	25.31	0.960	7.515	2.618	0.975
Fe-HBC	0.400	27.14	0.965	9.533	2.717	0.938

**Table 2:** Kinetics parameters based on pseudo-first-order and pseudo-second-order kinetics models.

Modified Biochar	Pseudo-First-Order Kinetic Equation			Pseudo-Second-Order Kinetic Equation		
	K_1_ (min^−1^)	Q_e1_ (mg g^−1^)	R^2^	K_2_ (g mg^−1^·min^−1^)	Q_e2_ (mg g^−1^)	R^2^
Fe-WBC	0.1471	29.172	0.8980	6.43 × 10^−3^	32.29	0.9633
Fe-BBC	0.2194	21.391	0.7401	1.12 × 10^−2^	27.86	0.9628
Fe-HBC	0.2096	25.519	0.8903	1.33 × 10^−2^	23.54	0.8915

## Data Availability

The data that support the findings of this study are available from the corresponding author upon request.

## References

[1] Qu J., Akindolie M.S., Feng Y., Jiang Z., Zhang G., Jiang Q., Deng F., Cao B., Zhang Y. (2020). One-pot hydrothermal synthesis of NaLa(CO_3_)_2_ decorated magnetic biochar for efficient phosphate removal from water: Kinetics, isotherms, thermodynamics, mechanisms and reusability exploration. Chem. Eng. J..

[2] Cao J.S., Zhao W.Y., Wang S.N., Xu R.Z., Hao L.S., Sun W. (2023). Effects of Calcium on Phosphorus Recovery from Wastewater by Vivianite Crystallization: Interaction and Mechanism Analysis. J. Environ. Chem. Eng..

[3] Le Moal M., Gascuel-Odoux C., Ménesguen A., Souchon Y., Étrillard C., Levain A., Moatar F., Pannard A., Souchu P., Lefebvre A. (2019). Eutrophication: A New Wine in an Old Bottle?. Sci. Total Environ..

[4] Wang B., Lian G.Q., Lee X.Q., Gao B., Li L., Liu T.Z., Zhang X.Y., Zheng Y.L. (2020). Phosphogypsum as a Novel Modifier for Distillers Grains Biochar Removal of Phosphate from Water. Chemosphere.

[5] Akram M., Gao B., Pan J., Khan R., Inam M.A., Xu X., Guo K., Yue Q. (2022). Enhanced Removal of Phosphate Using Pomegranate Peel-Modified Nickel-Lanthanum Hydroxide. Sci. Total Environ..

[6] Luo Y., Xie K., Feng Y., He Q., Zhang K., Shen S., Wang F. (2021). Synthesis of a La(OH)_3_ nanorod/walnut shell biochar composite for reclaiming phosphate from aqueous solutions. Colloids Surf. A Physicochem. Eng. Asp..

[7] Liu L., Zhang C., Chen S., Ma L., Li Y., Lu Y. (2022). Phosphate adsorption characteristics of La(OH)_3_-modified, canna-derived biochar. Chemosphere.

[8] Chen Q., Qin J., Sun P., Cheng Z., Shen G. (2018). Cow Dung-Derived Engineered Biochar for Reclaiming Phosphate from Aqueous Solution and Its Validation as Slow-Release Fertilizer in Soil-Crop System. J. Clean. Prod..

[9] Yang Q., Wang X., Luo W., Sun J., Xu Q., Chen F., Zhao J., Wang S., Yao F., Wang D. (2018). Effectiveness and Mechanisms of Phosphate Adsorption on Iron-Modified Biochars Derived from Waste Activated Sludge. Bioresour. Technol..

[10] Liu Y., Wang S.Y., Huo J.B., Zhang X.B., Wen H.T., Zhang D., Zhao Y., Kang D.J., Guo W.S., Ngo H.H. (2024). Adsorption recovery of phosphorus in contaminated water by calcium modified biochar derived from spent coffee grounds. Sci. Total Environ..

[11] Lürling M., Mackay E., Reitzel K., Spears B.M. (2016). Editorial—A critical perspective on geo-engineering for eutrophication management in lakes. Water Res..

[12] Jiang Y.H., Li A.Y., Deng H., Ye C.H., Wu Y.Q., Linmu Y.D., Hang H.L. (2019). Characteristics of Nitrogen and Phosphorus Adsorption by Mg-Loaded Biochar from Different Feedstocks. Bioresour. Technol..

[13] Feng Q.W., Chen M., Wu P., Zhang X.Y., Wang S.S., Yu Z.B., Wang B. (2022). Simultaneous Reclaiming Phosphate and Ammonium from Aqueous Solutions by Calcium Alginate-Biochar Composite: Sorption Performance and Governing Mechanisms. Chem. Eng. J..

[14] Huang Y.M., Lee X.Q., Grattieri M., Yuan M.W., Cai R., Macazo F.C., Minteer S.D. (2020). Modified Biochar for Phosphate Adsorption in Environmentally Relevant Conditions. Chem. Eng. J..

[15] He R.Z., Peng Z.Y., Lyu H.H., Huang H., Nan Q., Tang J.C. (2018). Synthesis and Characterization of an Iron-Impregnated Biochar for Aqueous Arsenic Removal. Sci. Total Environ..

[16] Shi W., Fu Y., Jiang W., Ye Y., Kang J., Liu D., Ren Y., Li D., Luo C., Xu Z. (2019). Enhanced phosphate removal by zeolite loaded with Mg–Al–La ternary (hydr)oxides from aqueous solutions: Performance and mechanism. Chem. Eng. J..

[17] Fu H., Yang Y., Zhu R., Liu J., Usman M., Chen Q., He H. (2018). Superior Adsorption of Phosphate by Ferrihydrite-Coated and Lanthanum-Decorated Magnetite. J. Colloid Interface Sci..

[18] Goscianska J., Ptaszkowska-Koniarz M., Frankowski M., Franus M., Panek R., Franus W. (2018). Removal of Phosphate from Water by Lanthanum-Modified Zeolites Obtained from Fly Ash. J. Colloid Interface Sci..

[19] Leng L., Huang H., Li H., Li J., Zhou W. (2019). Biochar Stability Assessment Methods: A Review. Sci. Total Environ..

[20] Zhao Z., Wang B., Feng Q., Chen M., Zhang X., Zhao R. (2023). Recovery of nitrogen and phosphorus in wastewater by red mud-modified biochar and its potential application. Sci. Total Environ..

[21] Ajmal Z., Muhmood A., Dong R.J., Wu S.B. (2020). Probing the Efficiency of Magnetically Modified Biomass-Derived Biochar for Effective Phosphate Removal. J. Environ. Manag..

[22] Cancelliere R., Mele P., Bartolucci L., Cordiner S., da Silva Freitas W., Mazzuca C., Mecheri B., Micheli L., Mulone V., Paialunga E. (2025). Mutual interaction of pyrolysis operating conditions and surface morphology for the electrochemical performance of biochar-modified screen-printed electrodes. J. Environ. Chem. Eng..

[23] Cancelliere R., Cosio T., Campione E., Corvino M., D’Amico M.P., Micheli L., Signori E., Contini G. (2023). Label-free electrochemical immunosensor as a reliable point-of-care device for the detection of Interleukin-6 in serum samples from patients with psoriasis. Front. Chem..

[24] Xiang W., Zhang X.Y., Chen J.J., Zou W.X., He F., Hu X., Tsang D.C.W., Ok Y.S., Gao B. (2020). Biochar Technology in Wastewater Treatment: A Critical Review. Chemosphere.

[25] Zhang M., Gao B. (2013). Removal of arsenic, methylene blue, and phosphate by biochar/AlOOH nanocomposite. Chem. Eng. J..

[26] Mishra P.C., Patel R.K. (2009). Use of agricultural waste for the removal of nitrate-nitrogen from aqueous medium. J. Environ. Manag..

[27] Cheng N., Wang B., Wu P., Lee X., Xing Y., Chen M., Gao B. (2021). Adsorption of emerging contaminants from water and wastewater by modified biochar: A review. Environ. Pollut..

[28] Yin Q., Ren H., Wang R., Zhao Z. (2018). Evaluation of Nitrate and Phosphate Adsorption on Al-Modified Biochar: Influence of Al Content. Sci. Total Environ..

[29] Gao N., Du W., Zhang M., Ling G., Zhang P. (2022). Chitosan-modified biochar: Preparation, modifications, mechanisms and applications. Int. J. Biol. Macromol..

[30] Kumar A., Singh E., Mishra R., Kumar S. (2022). Biochar as Environmental Armour and Its Diverse Role towards Protecting Soil, Water and Air. Sci. Total Environ..

[31] Mansoor S., Kour N., Manhas S., Zahid S., Wani O.A., Sharma V., Wijaya L., Alyemeni M.N., Alsahli A.A., El-Serehy H.A. (2021). Biochar as a tool for effective management of drought and heavy metal toxicity. Chemosphere.

[32] Gong H., Zhao L., Rui X., Hu J., Zhu N. (2022). A review of pristine and modified biochar immobilizing typical heavy metals in soil: Applications and challenges. J. Hazard. Mater..

[33] Yin Q., Wang R., Zhao Z. (2018). Application of Mg–Al-modified biochar for simultaneous removal of ammonium, nitrate, and phosphate from eutrophic water. J. Clean. Prod..

[34] Ahmed M.B., Zhou J.L., Ngo H.H., Guo W., Chen M. (2016). Progress in the preparation and application of modified biochar for improved contaminant removal from water and wastewater. Bioresour. Technol..

[35] Xu Z., Zhang C., Zhang C., Chen Z. (2023). Quantitative Evaluation on Phosphate Adsorption by Modified Biochar: A Meta-Analysis. Process Saf. Environ. Prot..

[36] Wu L., Wei C., Zhang S., Wang Y., Kuzyakov Y., Ding X. (2019). MgO-Modified Biochar Increases Phosphate Retention and Rice Yields in Saline-Alkaline Soil. J. Clean. Prod..

[37] Zhang M., Gao B., Yao Y., Xue Y.W., Inyang M. (2012). Synthesis of porous MgO-biochar nanocomposites for removal of phosphate and nitrate from aqueous solutions. Chem. Eng. J..

[38] Li X.Y., Xie Y.H., Jiang F., Wang B., Hu Q.L., Tang Y., Luo T., Wu T. (2020). Enhanced Phosphate Removal from Aqueous Solution Using Resourceable Nano-CaO_2_/BC Composite: Behaviors and Mechanisms. Sci. Total Environ..

[39] Cui L.Q., Noerpel M.R., Scheckel K.G., Ippolito J.A. (2019). Wheat Straw Biochar Reduces Environmental Cadmium Bioavailability. Environ. Int..

[40] Liu L., Fan S.S. (2018). Removal of cadmium in aqueous solution using wheat straw biochar: Effect of minerals and mechanism. Environ. Sci. Pollut. Res..

[41] Song Y., Wang F., Bian Y.R., Kengara F.O., Jia M.Y., Xie Z.B., Jiang X. (2012). Bioavailability assessment of hexachlorobenzene in soil as affected by wheat straw biochar. J. Hazard. Mater..

[42] Malik A. (2007). Environmental challenge vis a vis opportunity: The case of water hyacinth. Environ. Int..

[43] Gaurav G.K., Mehmood T., Cheng L., Klemes J.J., Shrivastava D.K. (2020). Water Hyacinth as a Biomass: A Review. J. Clean. Prod..

[44] Su J.Z., Guo Z.L., Zhang M.Y., Xie Y.M., Shi R., Huang X.F., Tuo Y., He X.H., Xiang P. (2024). Mn-Modified Bamboo Biochar Improves Soil Quality and Immobilizes Heavy Metals in Contaminated Soils. Environ. Technol. Innov..

[45] Chen D.G., Yu X.Z., Song C., Pang X.L., Huang J., Li Y.J. (2016). Effect of Pyrolysis Temperature on the Chemical Oxidation Stability of Bamboo Biochar. Bioresour. Technol..

[46] Li H.Y., Zhou C., Wang L.H., Yang F., Liang J.Y., Wang F., Li P.Y., Li C., Wu Z.G., Ren T.B. (2025). A Novel Eco-Friendly Bamboo-Based Composite Biochar for Effective Removing Oxytetracycline Hydrochloride. Adv. Compos. Hybrid Mater..

[47] Kayiranga A., Luo Z.X., Ndayishimiye J.C., Nkinahamira F., Cyubahiro E., Habumugisha T., Yan C.Z., Guo J.H., Zhen Z., Tuyishimire A. (2021). Insights into Thallium Adsorption onto the Soil, Bamboo-Derived Biochar, and Biochar Amended Soil in Pomelo Orchard. Biochar.

[48] Klein A.R., Bone S.E., Bakker E., Chang Z.Q., Aristilde L. (2019). Abiotic Phosphorus Recycling from Adsorbed Ribonucleotides on a Ferrihydrite-Type Mineral: Probing Solution and Surface Species. J. Colloid Interface Sci..

[49] Wang J.X., Zhang G.Q., Qiao S., Zhou J.T. (2021). Magnetic Fe^0^/Iron Oxide-Coated Diatomite as a Highly Efficient Adsorbent for Recovering Phosphorus from Water. Chem. Eng. J..

[50] Do Q.C., Ko S.O., Jang A., Kim Y., Kang S. (2020). Incorporation of Iron (Oxyhydr)oxide Nanoparticles with Expanded Graphite for Phosphorus Removal and Recovery from Aqueous Solutions. Chemosphere.

[51] Liang D.H., Chang J.F., Wu Y., Wang S., Wang X., Ren N.Q., Li N. (2024). The Screening of Iron Oxides for Long-Term Transformation into Vivianite to Recover Phosphorus from Sewage. Water Res..

[52] Vikrant K., Kim K.H., Ok Y.S., Tsang D.C.W., Tsang Y.F., Giri B.S., Singh R.S. (2018). Engineered/Designer Biochar for the Removal of Phosphate in Water and Wastewater. Sci. Total Environ..

[53] Goh K.H., Lim T.T., Dong Z. (2008). Application of Layered Double Hydroxides for Removal of Oxyanions: A Review. Water Res..

[54] Li J., Li B., Huang H., Lv X., Zhao N., Guo G., Zhang D. (2019). Removal of Phosphate from Aqueous Solution by Dolomite-Modified Biochar Derived from Urban Dewatered Sewage Sludge. Sci. Total Environ..

[55] Liu H.B., Shan J.H., Chen Z.B., Lichtfouse E. (2021). Efficient Recovery of Phosphate from Simulated Urine by Mg/Fe Bimetallic Oxide Modified Biochar as a Potential Resource. Sci. Total Environ..

[56] Zhang T., Wu X.S., Shaheen S.M., Zhao Q., Liu X.J., Rinklebe J., Ren H.Q. (2020). Ammonium nitrogen recovery from digestate by hydrothermal pretreatment followed by activated hydrochar sorption. Chem. Eng. J..

[57] Yu J., Li X.D., Wu M., Lin K., Xu L.H., Zeng T., Shi H.X., Zhang M. (2022). Synergistic role of inherent calcium and iron minerals in paper mill sludge biochar for phosphate adsorption. Sci. Total Environ..

[58] Islam M.S., Kwak J.H., Nzediegwu C., Wang S.Y., Palansuriya K., Kwon E.E., Naeth M.A., El-Din M.G., Ok Y.S., Chang S.X. (2021). Biochar Heavy Metal Removal in Aqueous Solution Depends on Feedstock Type and Pyrolysis Purging Gas. Environ. Pollut..

[59] Wang S.D., Kong L.J., Long J.Y., Su M.H., Diao Z.H., Chang X.Y., Chen D.Y., Song G., Shih K.M. (2018). Adsorption of Phosphorus by Calcium-Flour Biochar: Isotherm, Kinetic and Transformation Studies. Chemosphere.

[60] Xue P., Hou R., Fu Q., Li T., Wang J., Zhou W., Shen W., Su Z., Wang Y. (2023). Potentially Migrating and Residual Components of Biochar: Effects on Phosphorus Adsorption Performance and Storage Capacity of Black Soil. Chemosphere.

[61] Yin H.B., Kong M., Fan C.X. (2013). Batch Investigations on P Immobilization from Wastewaters and Sediment Using Natural Calcium Rich Sepiolite as a Reactive Material. Water Res..

[62] Palansooriya K.N., Kim S., Igalavithana A.D., Hashimoto Y., Choi Y.E., Mukhopadhyay R., Sarkar B., Ok Y.S. (2021). Fe(III) loaded chitosan-biochar composite fibers for the removal of phosphate from water. J. Hazard. Mater..

[63] Liu Y. (2008). New insights into pseudo-second-order kinetic equation for adsorption. Colloids Surf. A Physicochem. Eng. Asp..

[64] Fu X., Wang P., Wu J., Zheng P., Wang T., Li X., Ren M. (2022). Hydrocotyle Vulgaris Derived Novel Biochar Beads for Phosphorus Removal: Static and Dynamic Adsorption Assessment. J. Environ. Chem. Eng..

[65] Wang Z.H., Huang Z.L., Zheng B.Y., Wu D.S., Zheng S.L. (2022). Efficient Removal of Phosphate and Ammonium from Water by Mesoporous Tobermorite Prepared from Fly Ash. J. Environ. Chem. Eng..

[66] Xiong W.P., Tong J., Yang Z.H., Zeng G.M., Zhou Y.Y., Wang D.B., Song P.P., Xu R., Zhang C., Cheng M. (2017). Adsorption of Phosphate from Aqueous Solution Using Iron-Zirconium Modified Activated Carbon Nanofiber: Performance and Mechanism. J. Colloid Interface Sci..

[67] Lv X.S., Zhang Y.L., Fu W.Y., Cao J.Z., Zhang J., Ma H.B., Jiang G.M. (2017). Zero-valent iron nanoparticles embedded into reduced graphene oxide-alginate beads for efficient chromium (VI) removal. J. Colloid Interface Sci..

[68] Qiu Y.P., Cheng H.Y., Xu C., Sheng D. (2008). Surface characteristics of crop-residue-derived black carbon and lead(II) adsorption. Water Res..

[69] Hu X.Y., Peng X.J., Kong L.H., Zhu F. (2019). The Mechanism for Promoted Oxygenation of V(IV) by Goethite: Positive Effect of Surface Hydroxyl Groups. J. Hazard. Mater..

[70] Machala L., Tucek J., Zboril R. (2011). Polymorphous Transformations of Nanometric Iron(III) Oxide: A Review. Chem. Mater..

[71] Ristic M., Opacak I., Stajdohar J., Music S. (2015). The influence of CTAB and gum arabic on the precipitation of α-FeOOH in a highly alkaline medium. J. Mol. Struct..

